# Posterior reversible encephalopathy syndrome during epidural labor analgesia: a case report

**DOI:** 10.1186/s40981-025-00809-5

**Published:** 2025-08-23

**Authors:** Sadamu Sugimoto, Misako Shimizu, Mariko Takebe, Kousou Matsuura, Tomonori Takazawa

**Affiliations:** 1 Department of Anesthesiology, University of Toyama, , 2630 Sugitani, Toyama, Toyama 930-0194 Japan; 2 Department of Anesthesiology, Toyama City Hospital, 2-1 Hokubu-Cho, Toyama, Toyama 939-8511 Japan

**Keywords:** Posterior reversible encephalopathy syndrome, Preeclampsia, Eclampsia, Epidural labor analgesia, Amnesia

## Abstract

**Background:**

Posterior reversible encephalopathy syndrome (PRES) often presents with a wide range of neurological symptoms, and atypical manifestations can complicate its diagnosis. We report a rare case of peripartum PRES presenting with profound transient retrograde amnesia and orofacial automatisms, notably in the absence of generalized seizures.

**Case presentation:**

A 29-year-old primigravida developed sustained hypertension during labor. Immediately postpartum, she experienced visual disturbances, followed by altered consciousness and lip-smacking movements. She subsequently developed profound but transient retrograde amnesia, including loss of autobiographical memory. Brain magnetic resonance imaging (MRI) revealed characteristic findings of PRES in the bilateral parieto-occipital lobes, leading to a diagnosis of PRES secondary to preeclampsia.

**Discussion:**

This case highlights that peripartum PRES can present with atypical neurological symptoms, such as transient global amnesia and facial automatisms, even in the absence of typical eclamptic seizures. Such presentations warrant a high index of suspicion and prompt brain MRI to ensure accurate diagnosis and timely intervention.

## Background

Posterior reversible encephalopathy syndrome (PRES), which occurs primarily due to edema in the posterior cerebral region, presents with acute neurological symptoms and distinctive findings on neuroimaging [1, 2]. While PRES has a favorable prognosis and its symptoms are typically reversible, delayed diagnosis or treatment can lead to permanent neurological sequelae or even death [3, 4]. The primary theory regarding the pathogenesis of PRES suggests that the condition occurs when severe hypertension overwhelms the brain’s autoregulatory capacity for blood flow regulation. This leads to endothelial dysfunction, increased vascular permeability, and subsequent vasogenic edema [1, 2]. Consequently, PRES presents with characteristic hyperintense lesions on magnetic resonance imaging (MRI) [1, 2].

In obstetric patients, PRES is closely associated with hypertensive disorders of pregnancy, especially preeclampsia and eclampsia. Most eclamptic patients exhibit imaging findings consistent with PRES [5–7], leading some to consider PRES and eclampsia as part of the same disease spectrum [6, 8]. However, PRES can present with a wide range of atypical symptoms, making diagnosis challenging.

This report details the case of a patient who developed acute central nervous system symptoms in the immediate postpartum period and was subsequently diagnosed with PRES.

## Case presentation

A 29-year-old primigravida was admitted for labor management at 40 weeks and 1 day of gestation. The initial plan was for a conventional vaginal delivery without neuraxial analgesia. Her medical history was unremarkable. Her pregnancy had been uneventful, with maintenance of normotension and no signs of preeclampsia before admission. On admission, her initial blood pressure was 141/73 mmHg. Other physical findings were unremarkable, and no edema was observed. Labor progression was slow due to uterine inertia. The patient reported severe labor pain, and although pentazocine was administered, its analgesic effect was insufficient.

**Fig. 1:**
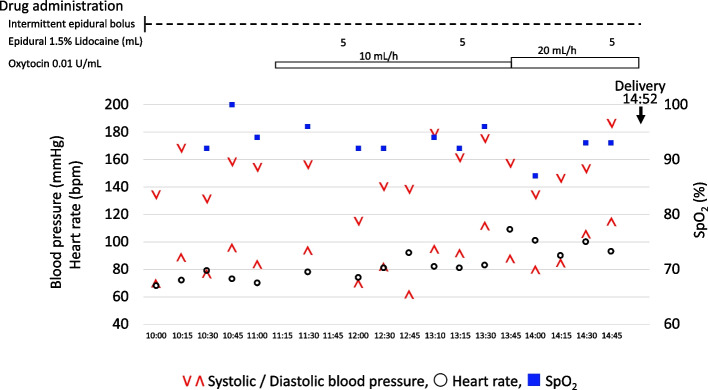
Clinical course before delivery. The timeline displays key clinical events, drug administrations, and vital signs in relation to the time of delivery. V: systolic blood pressure (mmHg); Λ: diastolic blood pressure (mmHg); ○: heart rate (bpm); ■: SpO2 (%), percutaneous oxygen saturation

On the morning of hospital day 2, the patient’s labor was progressing slowly, and she was experiencing severe pain and significant maternal fatigue. Therefore, at 9:00 AM, the decision was made to proceed with labor augmentation and provide neuraxial analgesia, prompting the first consultation with the anesthesiology service. At the time of the consultation, the patient did not meet the diagnostic criteria for hypertensive disorders of pregnancy (HDP), as her blood pressure elevations had been intermittent rather than sustained. Continuous epidural analgesia was initiated at 10:15 AM to manage her pain and fatigue, followed by the commencement of an oxytocin infusion for labor augmentation at 11:00 AM (Fig. 1). The epidural regimen consisted of intermittent boluses (5 mL every 45 min) of 0.1% ropivacaine with fentanyl (2 µg/mL) and patient-controlled epidural analgesia (PCEA) boluses (5 mL, 20-min lockout). Additional 5 mL boluses of 1.5% lidocaine were administered for breakthrough pain. Although the patient achieved adequate analgesia, it became insufficient over time, necessitating frequent self-administration via the PCEA and supplemental lidocaine boluses. Her blood pressure gradually increased, fluctuating between 140–180 and 70–90 mmHg during contractions and 110–130/60–80 mmHg between them (Fig. 1). Four hours after initiating epidural analgesia, the patient underwent vacuum-assisted vaginal delivery with the Kristeller maneuver. The newborn had Apgar scores of 9 and 10 at 1 and 5 min, respectively.

**Fig. 2:**
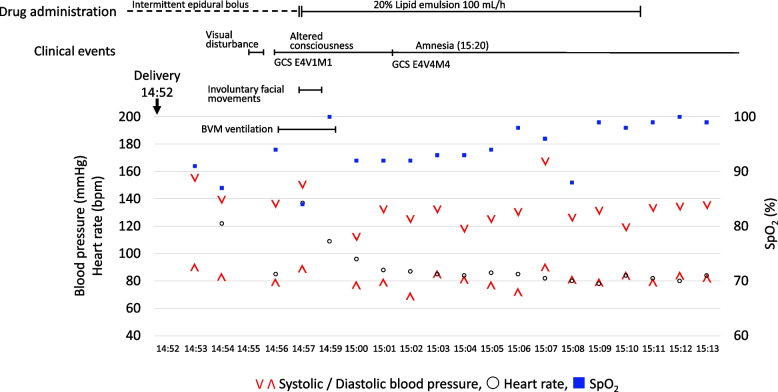
Clinical course in the immediate postpartum period. The timeline displays the key clinical events (visual disturbance, altered consciousness, amnesia), drug administrations, and vital signs. V: systolic blood pressure (mmHg); Λ: diastolic blood pressure (mmHg); ○: heart rate (bpm); ■: SpO2 (%), percutaneous oxygen saturation; GCS: Glasgow coma scale (E, eye-opening; V, verbal response; M, motor response); BVM ventilation: bag-valve-mask ventilation

Three minutes postpartum, the patient complained of blurred vision. One minute later, she developed a sudden decline in consciousness, with a Glasgow Coma Scale (GCS) score of E4V1M1. Her oxygen saturation transiently dropped to 60–70%, requiring bag-valve-mask ventilation. Suspecting local anesthetic systemic toxicity (LAST), the epidural infusion was immediately discontinued, and intravenous lipid emulsion therapy was initiated. Although generalized convulsions were not observed, the patient exhibited persistent involuntary facial movements, including blinking and lip-smacking. Her level of consciousness recovered to GCS E4V4M4 approximately 4 min later. Despite the improvement in consciousness, she displayed profound retrograde amnesia. The memory impairment was profound, manifesting as complete disorientation to person, place, and time, including the inability to recall her name and the events of her pregnancy and delivery. This severe amnestic state entirely resolved within 20 min (Fig. 2).

**Fig. 3:**
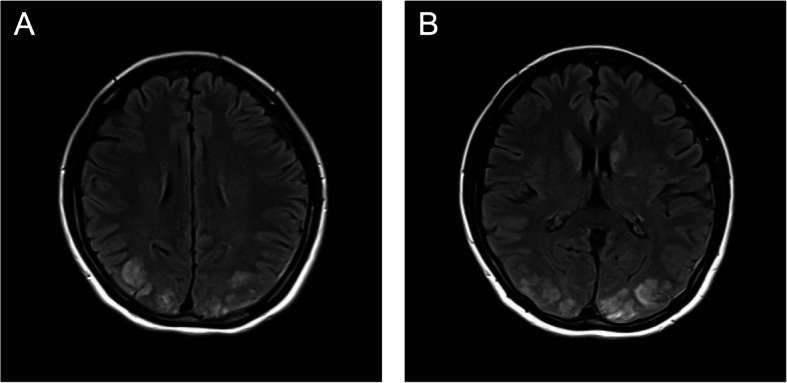
Magnetic resonance imaging (MRI) findings. Axial FLAIR images show hyperintensities in the parietal lobes (A) and occipital lobes (B) bilaterally. FLAIR: fluid-attenuated inversion recovery

Subsequently, a complete diagnostic workup was performed. Given the central nervous system symptoms, an eclampsia-related pathology was suspected, and a continuous intravenous infusion of magnesium sulfate was initiated 30 min postpartum. Urinalysis revealed a protein-to-creatinine ratio of 1.0. The serum lidocaine concentration, measured from a blood sample drawn at the same time, was reported the following day as being within the normal range. Her other laboratory findings are summarized in Table 1. Abnormal findings included leukocytosis, elevated C-reactive protein, hypoalbuminemia, hyperuricemia, and hyperammonemia. Mild elevations in AST, LDH, and CK were also observed. The coagulation profile was notable for elevated FDP and D-dimer with low antithrombin III levels, and blood gas analysis indicated metabolic acidosis. A non-contrast cranial computed tomography (CT) scan showed no evidence of intracranial hemorrhage. Subsequently, brain MRI revealed scattered, symmetric hyperintense signals on FLAIR sequences, predominantly in the parietal and occipital lobes bilaterally (Fig. 3). Based on these collective findings, the patient was diagnosed with PRES secondary to preeclampsia.

**Table 1:** Laboratory findings 30 min postpartum

Category	Test item	Result	Unit
**Blood gas analysis**			
	pH	7.437	
	PCO_2_	23.4	mmHg
	PO_2_	193	mmHg
	HCO_3_^–^	15.5	mmol/L
	BE	−7.3	mmol/L
	MetHb	2.9	%
**Complete blood count**			
	WBC	21.6	× 10^3^/µL
	Neutrophil	89	%
	Lymphocyte	7	%
	RBC	3.91	× 10⁶/µL
	Hb	12.1	g/dL
	Hct	33.1	%
	Plt	316	× 10^3^/µL
**Coagulation profile**			
	PT-INR	0.9	
	APTT	30.8	sec
	Fibrinogen	470	mg/dL
	FDP	23.8	µg/mL
	D-dimer	11.4	µg/mL
	AT III	57	%
**Biochemistry**			
	AST	45	U/L
	ALT	18	U/L
	LDH	313	U/L
	Creatinine	0.84	mg/dL
	eGFR	66	mL/min/1.73 m^2^
	BUN	10	mg/dL
	Uric acid	7	mg/dL
	Total protein	5.2	g/dL
	Albumin	2.4	g/dL
	Sodium	134	mEq/L
	Potassium	4	mEq/L
	Calcium	8.5	mg/dL
	Chloride	103	mEq/L
	Magnesium	2.1	mg/dL
	Ammonia	180	µg/dL
**Inflammatory markers and others**			
	CRP	3.74	mg/dL
	CK	609	U/L

*PCO*_*2*_ Partial pressure of carbon dioxide, *PO*_*2*_ Partial pressure of oxygen, *HCO*_*3*_^*–*^ Bicarbonate, *BE* Base excess, *MetHb* Methemoglobin, *WBC* White blood cell, *RBC* Red blood cell, *Hb* Hemoglobin, *Hct* Hematocrit, *Plt* Platelet count, *PT-INR* Prothrombin time-international normalized ratio, *APTT* Activated partial thromboplastin time, *FDP* Fibrinogen degradation products, *AT III* Antithrombin III, *AST* Aspartate aminotransferase, *ALT* Alanine aminotransferase, *LDH* Lactate dehydrogenase, *eGFR* Estimated glomerular filtration rate, *BUN* Blood urea nitrogen, *CRP* C-reactive protein, *CK* Creatine kinase

**Fig. 4:**
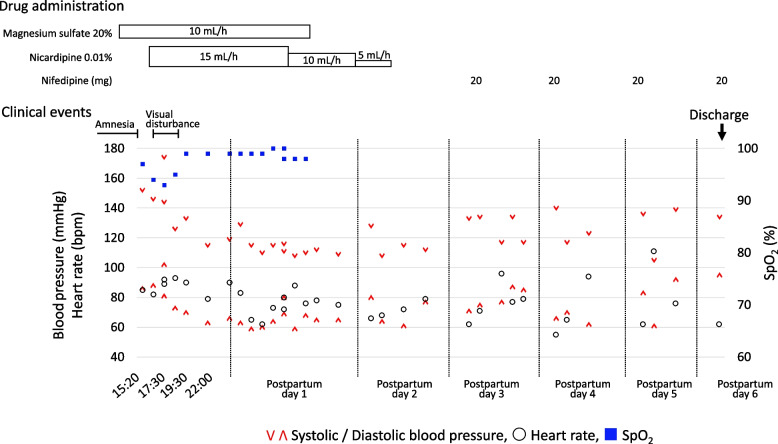
Clinical course and management during the extended postpartum period. The chart illustrates the gradual tapering of intravenous nicardipine and the transition to oral nifedipine beginning on postpartum day 3, in parallel with the normalization of vital signs and subsequent discharge. V: systolic blood pressure (mmHg); Λ: diastolic blood pressure (mmHg); ○: heart rate (bpm); ■: SpO2 (%), percutaneous oxygen saturation

Following delivery, the patient was admitted to the high care unit (HCU) for close monitoring (Fig. 4). At 2.5 h postpartum, her blood pressure increased again to 174/102 mmHg, and her visual disturbances recurred. Continuous intravenous nicardipine was initiated. The patient reported distorted perceptions of faces and objects, and visual field testing revealed central visual field defects in both eyes. These symptoms resolved completely within two hours with blood pressure control. On postpartum day 1, her blood pressure normalized, and magnesium sulfate was discontinued. On postpartum day 1, as her blood pressure stabilized and she remained neurologically asymptomatic, the continuous nicardipine infusion was stopped and replaced with oral nifedipine, and she was transferred from the HCU to the general ward. Her clinical course was uneventful thereafter, and she was discharged on postpartum day 6 without neurological sequelae. Her blood pressure remained stable during outpatient follow-up, and oral nifedipine was discontinued 35 days after discharge. A follow-up brain MRI was not performed as the patient was clinically asymptomatic.

## Discussion

Our patient was diagnosed with PRES secondary to preeclampsia based on sustained hypertension during labor, proteinuria, and characteristic MRI findings.

The differential diagnosis of acute peripartum neurological symptoms is broad and includes eclampsia, cerebrovascular events, hemolysis, elevated liver enzymes, and low platelet count (HELLP) syndrome, and amniotic fluid embolism [9, 10]. Given the use of continuous epidural analgesia in this case, LAST was also considered a primary differential diagnosis.

While eclampsia was indeed a primary consideration in the acute setting, two key factors prompted us to broaden the differential diagnosis. First, the patient did not experience a generalized tonic–clonic seizure, which is typically regarded as a hallmark of eclampsia in clinical practice in Japan. Second, the neurological symptoms occurred during continuous epidural analgesia, raising concern for local anesthetic systemic toxicity (LAST), which needed to be urgently ruled out. These conditions were systematically excluded for the following reasons. LAST was initially prioritized due to its temporal association with epidural analgesia. However, the patient's symptoms did not align with the classic features of LAST, such as perioral numbness, generalized seizures, arrhythmias, and cardiovascular collapse. Furthermore, serum lidocaine concentration measured shortly after symptom onset was within the normal range, further ruling out this possibility. In cases like ours, where atypical neurological symptoms predominate, distinguishing LAST from conditions such as PRES or eclampsia can be challenging, as has been previously reported in the literature [11].

While cerebrovascular events were also considered, they were deemed less likely due to the transience of the focal neurological deficits and altered consciousness, as well as the lack of evidence of hemorrhage, infarction, or cerebral venous thrombosis on cranial CT and MRI. HELLP syndrome was ruled out, as the patient did not present with characteristic liver dysfunction or thrombocytopenia (Table 1). Similarly, amniotic fluid embolism was excluded due to the absence of typical features, such as disseminated intravascular coagulation (DIC), circulatory collapse, and severe respiratory distress.

Although formal diagnostic criteria for Posterior Reversible Encephalopathy Syndrome (PRES) have not been established, a clinical framework for its diagnosis has been proposed. This framework defines PRES by a triad of findings: (1) acute-onset neurological symptoms, (2) vasogenic edema on neuroimaging, and (3) reversibility of the clinical and/or radiological findings [5].

Our patient’s case satisfied all three criteria. She presented with acute neurological symptoms, including visual disturbances, altered consciousness, and transient global amnesia. Neuroimaging revealed characteristic vasogenic edema in the bilateral parieto-occipital lobes (Fig. 3). Finally, the rapid resolution of her symptoms following blood pressure control demonstrated clinical reversibility, strongly supporting the diagnosis of PRES secondary to preeclampsia.

Although this case was diagnosed as PRES secondary to preeclampsia, the clinical course deviated from that of typical eclampsia in two important aspects. First, the absence of a generalized tonic–clonic seizure was atypical for eclampsia-related PRES. While generalized tonic–clonic seizures are the most common presentation, focal seizures are also known to occur, albeit infrequently [12, 13]. In this case, a neurologist considered the lip-smacking observed during the episode of acute impaired consciousness to represent one of two possibilities, both of which are rare manifestations of PRES: facial clonus or orofacial automatisms as part of a focal seizure. This uncommon seizure presentation contributed to the initial consideration of an alternative diagnosis beyond eclampsia. Second, the presence of transient yet profound global amnesia was another distinguishing feature. The patient temporarily lost significant autobiographical memories, including those about her marriage, pregnancy, and even her own name. Although amnesia is a rare complication, similar cases of transient global amnesia associated with PRES have been reported, showing comparable amnestic symptoms and rapid resolution following blood pressure control [14]. Characteristically, the patient's amnesia occurred as part of the acute presentation of PRES, was not accompanied by delirium or evidence of structural brain damage, and resolved rapidly with blood pressure control. This highlights the diverse and often atypical clinical manifestations of PRES.

In retrospect, the patient’s initial complaint of visual disturbance might have represented an important prodromal symptom of PRES. Although the nature of visual symptoms can vary, they are a well-known early sign of PRES and often precede other neurological abnormalities [1, 2, 5]. In this case, the patient reported visual disturbances prior to both episodes of impaired consciousness, with the second episode, in particular, being consistent with the typical features of cortical blindness. This clinical course suggests vasogenic edema in the occipital lobes, consistent with the bilateral occipital FLAIR hyperintensities observed on MRI.

This case highlights the importance of maintaining a high index of suspicion for PRES in the peripartum setting, even in the absence of classic features such as sustained hypertension or generalized seizures. Transient and atypical neurological symptoms, such as orofacial automatisms and reversible retrograde amnesia, might serve as early indicators of PRES and should prompt timely neuroimaging and appropriate management.

In conclusion, we report a rare case of peripartum-onset PRES presenting with atypical neurological features, including orofacial automatisms and transient retrograde amnesia. Clinicians should consider PRES in the differential diagnosis of acute neurological changes during labor and in the immediate postpartum period, even in the absence of typical manifestations.

## Data Availability

The datasets used and analyzed during the current study are available from the corresponding author upon reasonable request.

## Abbreviations

ALT: Alanine aminotransferase
APTT: Activated partial thromboplastin time
AST: Aspartate aminotransferase
AT III: Antithrombin III
BE: Base excess
BUN: Blood urea nitrogen
BVM: Bag-valve-mask
CK: Creatine kinase
CRP: C-reactive protein
CT: Computed tomography
DIC: Disseminated intravascular coagulation
eGFR: Estimated glomerular filtration rate
FDP: Fibrinogen degradation products
FLAIR: Fluid-attenuated inversion recovery
GCS: Glasgow coma scale
Hb: Hemoglobin
HCO_3_^–^: Bicarbonate
Hct: Hematocrit
HELLP: Hemolysis, elevated liver enzymes, and low platelet count
LAST: Local anesthetic systemic toxicity
LDH: Lactate dehydrogenase
MetHb: Methemoglobin
MRI: Magnetic resonance imaging
PCEA: Patient-controlled epidural analgesia
PCO_2_: Partial pressure of carbon dioxide
Plt: Platelet count
PO_2_: Partial pressure of oxygen
PRES: Posterior reversible encephalopathy syndrome
PT-INR: Prothrombin time-international normalized ratio
RBC: Red blood cell
SpO_2_: Percutaneous oxygen saturation
WBC: White blood cell
